# Assessment of Pulmonary Metastasis in Differentiated Thyroid Carcinoma: Value of HRCT Correlation with Functional Imaging

**DOI:** 10.1055/s-0043-1764307

**Published:** 2023-04-28

**Authors:** Ashwini Kalshetty, Sandip Basu

**Affiliations:** 1 Radiation Medicine Centre (BARC), Tata Memorial Hospital Annexe, Parel, Mumbai, Maharashtra, India; 2 Homi Bhabha National Institute, Mumbai, Maharashtra, India

**Keywords:** differentiated thyroid carcinoma, pulmonary metastasis, high resolution CT (HRCT), multiple detector computed tomography (MDCT), radioiodine scintigraphy, chest X-ray, SPECT-CT

## Abstract

Pulmonary metastases in thyroid carcinoma demonstrates varying imaging characteristics and disease biology and the outcome. The valuable complimentary role of high-resolution CT (HRCT) in conjunction with functional imaging such as radioiodine scan has been discussed and illustrated in this review along with the varied clinical and imaging presentations of lung metastases from differentiated thyroid cancer (DTC). A multi-modality patient-specific diagnostic approach and awareness about the atypical presentations helps in early identification as well as effective management of these patients, and especially in certain situations that could need multi-disciplinary management. While HRCT of the lungs as an added tool provides detailed visualization of the lung parenchyma, in the era of hybrid imaging, the routine adoption of SPECT-CT in patients with pulmonary metastases (in diagnostic or post-treatment settings) could provide equivalent or even incremental information from further management viewpoint.

## Introduction

Pulmonary metastases from thyroid carcinoma are usually asymptomatic, but have inferior outcome compared with localized disease. As personalized management in oncological practice develops, new and patient-specific diagnostic and treatment algorithms are being introduced at a rapid pace. The current American Thyroid Association (ATA) guidelines addressing the entire spectrum of thyroid cancer emphasizes on such patient-specific management of differentiated thyroid carcinoma (DTC). Different subsets of patients of pulmonary metastasis with varied clinical behavior is frequently encountered in routine clinical practice. Some patients are administered higher radioiodine doses and as such they require multiple doses as recommended by the ATA guidelines, the dose-limiting factors being radiation-induced fibrosis or reaching the maximum bone-marrow dose. 1

Studies have documented various patterns of radioiodine uptake in lung metastases originating from differentiated thyroid carcinoma (DTC). They are usually classified as bilateral diffuse, focal or ^131^ I-non-avid. 2 Similarly, the pulmonary metastases have also been classified based on the appearance on anatomical imaging modalities such as micronodular or macronodular (characterized by the presence of appreciable on chest X-ray or else). In recent years, with the of HRCT, there have also been introduction of terminologies, such as fine miliaric or nodular. 3 Early literature evaluated lung metastasis from DTC on HRCT and showed that the distribution of nodules is predominantly in lower zones and peripheral in location. 4 The ATA guidelines consider micrometastases as nodules less than 2 mm in size. 1 One gray zone area in such cases of micronodular disease is when the patients present with non-iodine concentrating asymptomatic tiny pulmonary metastases with low Tg and low clinical suspicion. The ATA recommendations are that the systemic therapy should be justified and reserved in such scenarios, with requirement of confirming the diagnosis by biopsy before therapeutic decision making, as pulmonary nodules attributable to benign causes are common in practice.

Additionally, monitoring response, follow-up, and disease prognosis in patients with pulmonary metastasis are important scenarios where evidence is inadequate at present, which modality is optimal for assessment. However, most studies suggest that those lung metastases detected only on functional imaging with initial low serum thyroglobulin (Tg) value have better prognosis (higher response to radioiodine therapy and disease-specific survival rates) than those with overt morphological changes and significantly high serum Tg values. 5 6 Age less than 45 years, high risk and aggressive histopathological features, distant metastases other than lungs and macronodular lung lesions are designated as predictors of poor outcome. 6 It is generally accepted that the volume of FDG positive metastatic disease (that is applicable to lung metastases also) is a determinant of prognosis over real time. 7

In this pictorial essay, we have endeavored to demonstrate the spectrum of lung metastases in thyroid carcinoma on various imaging modalities, emphasizing on the areas for multimodality assessment and also present gray zones in their clinical management.

## Case Illustrations

### Various Patterns Uptake on Diagnostic Radioiodine Scan

**Fig. 1:**
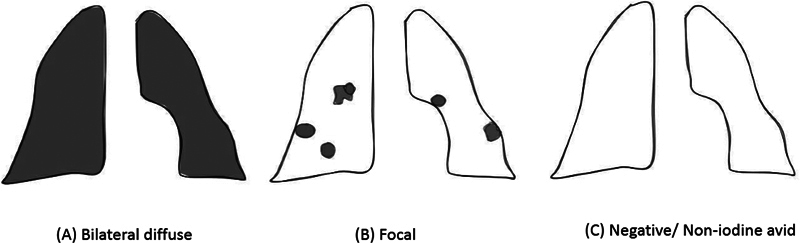
Schematic representation of various patterns of radioiodine uptake encountered on planar gamma camera imaging in patients with lung metastases from DTC.

The various patterns of radioiodine uptake observed in pulmonary metastasis include ( Fig. 1 ):

1. Diffuse
2. Focal
3. Negative

**Fig. 2:**
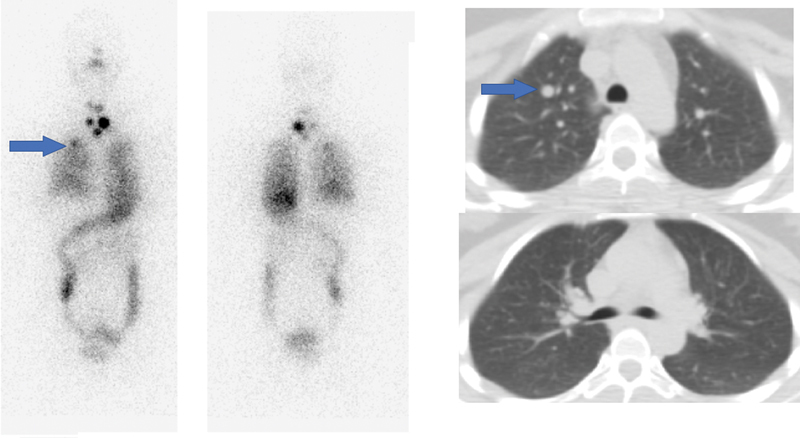
Diffusely increased tracer radioiodine uptake is noted in both lungs with a focally increased tracer uptake in the upper lobe of the right lung ( *blue arrow* in the left panel), corresponding to the 1.1 cm nodule observed on HRCT chest ( *right panel* ).

**Fig. 3:**
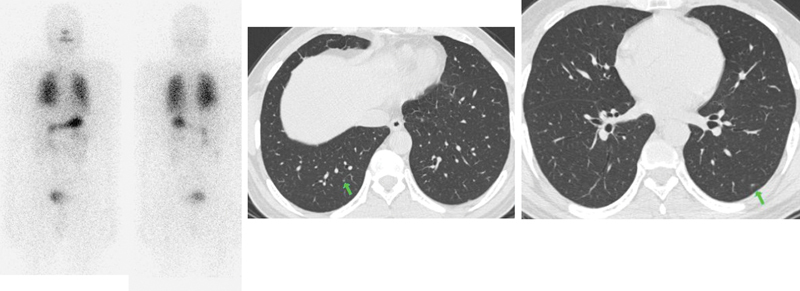
Diffusely increased radioiodine uptake is seen in both lungs with tiny nodules seen on HRCT ( *green arrow* ) of size of 1 to 2 mm.

**Fig. 4:**
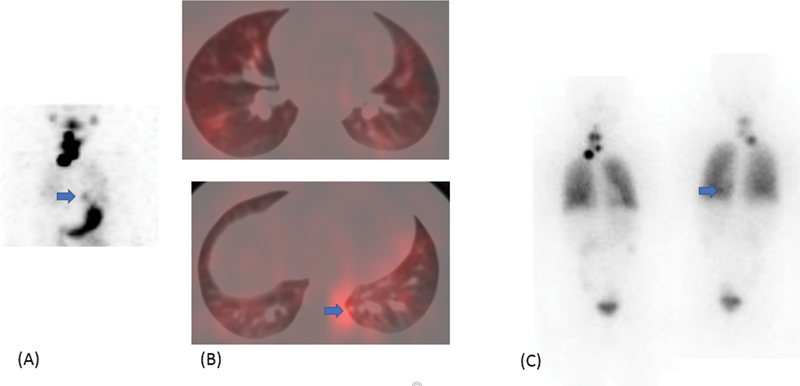
( **A** ) Multifocal ^131^ I uptake in the neck with diffuse uptake in both the lungs. Focal increased uptake is seen in the left lower zone ( *blue arrow* ) in SPECT MIP, which is difficult to perceive on planar images. ( **B** ) Fused SPECT CT shows diffuse uptake in bilateral lungs with focal increased tracer uptake in the left lung lower lobe ( *blue arrow* ), corresponding to the 4 × 4 mm nodule seen on CT. ( **C** ) Post-therapy scan shows multifocal ^131^ I uptake in the neck and diffuse uptake in both lungs. Focal increased uptake is seen in the left lower zone ( *blue arrow* ), which is difficult to perceive on planar images.

**Fig. 5:**
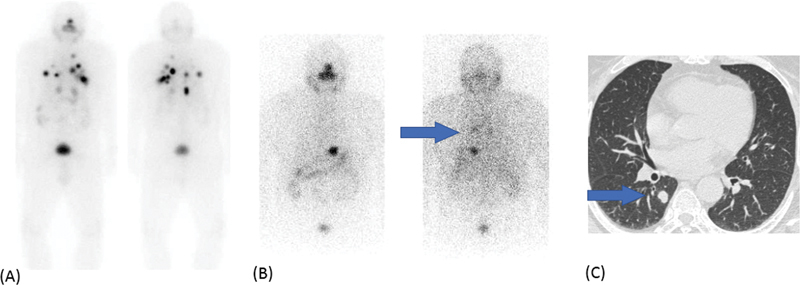
Multiple foci of increased radioiodine uptake are ( **A** ) noted in bilateral lungs. His follow-up diagnostic scan ( **B** ) showed resolution of most of the lung lesions with faint tracer uptake seen in lower lobe of left zones on posterior image ( *blue arrow* ). HRCT ( **C** ) shows a 0.8 cm nodule in the right lung lower lobe ( *blue arrow* ), corresponding to the focal uptake in the ^131^ I planar scan.

**Fig. 6:**
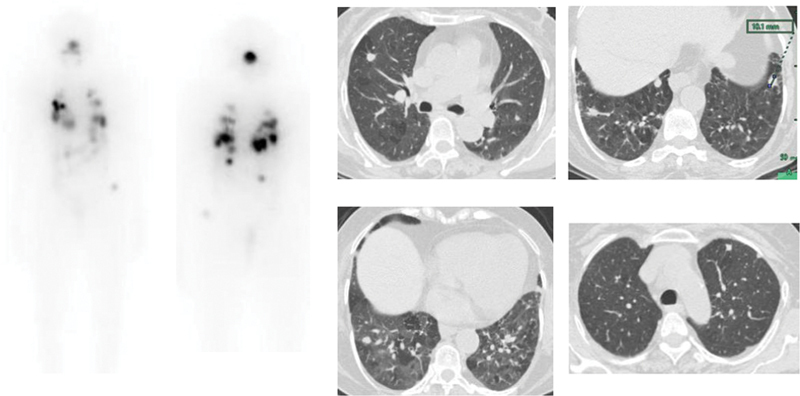
Patchy multi-focal uptake observed in bilateral lungs on ^131^ I WBS ( *left panel* ), along with iodine avid skeletal lesions. Also noted are the left cerebellar and left iliac lesions. HRCT images ( *right panel* ) shows multiple varying sized lung nodules, largest up to 1 cm.

**Fig. 7:**
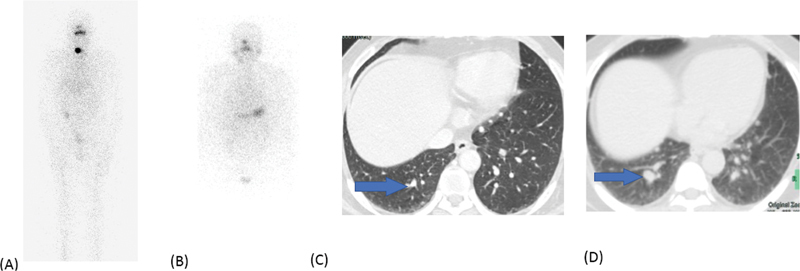
Initial diagnostic ^131^ I scan ( **A** ) shows focal increased uptake in the neck and tiny nodules in lungs. Follow-up scan after 8 months after radioiodine therapy shows resolution of ^131^ I avid neck lesion with increase in size of lung nodules (maximum 1.3 × 1.1 cm), none of which were positive on radioiodine scan.

**Fig. 8:**
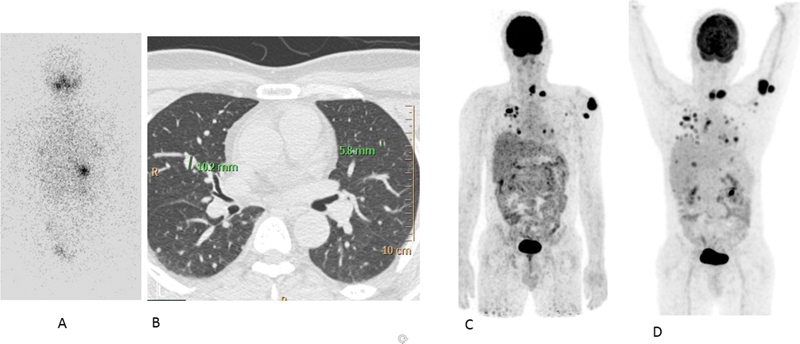
Follow-up ^131^ I scan ( **A** ) showing no appreciable tracer uptake in the chest with multiple nodules on HRCT (maximum size of 1 × 1 cm) ( **B** ). FDG PET CT ( **C, D** ) at 2 years shows disease progression with increase in the extent and metabolic activity of multiple lung nodules and soft tissue deposits in the neck and arm.

**Fig. 9:**
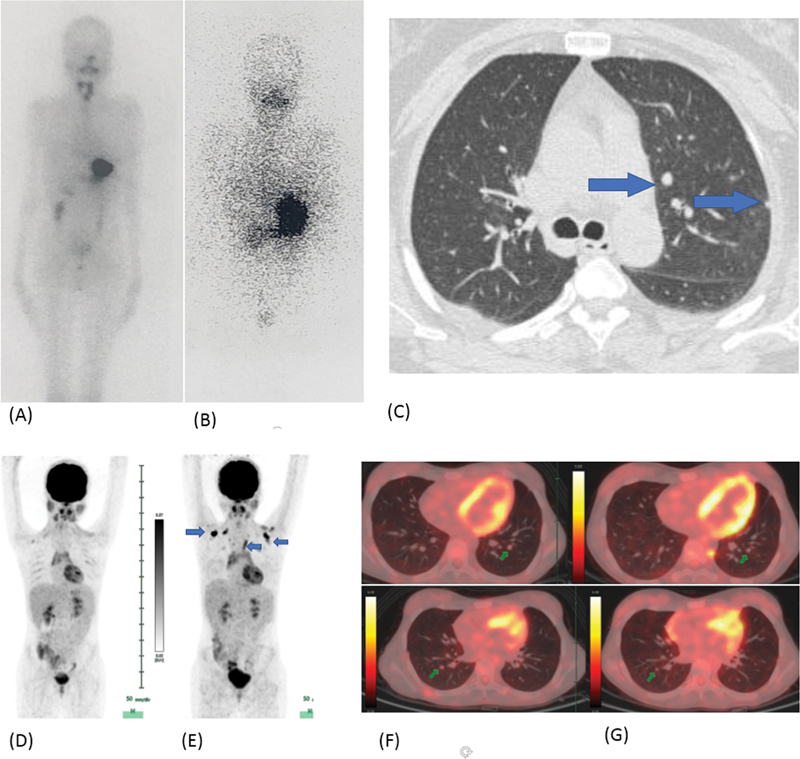
( **A** – **C** ) Initial ^131^ I uptake in the neck that resolves on follow-up ^131^ I imaging but with nodules on HRCT ( *blue arrow* ), suggesting ^131^ I non-avid lung lesions. ( **D** – **G** ) MIPs ( *left panel* ) of FDG PET-CT done at 1 year interval ( **D, E** ). The second PET-CT ( **E** ) on the right showed increased brown fat uptake ( *arrows* ). Physiologic thymic uptake is seen in both scans. The transaxial fused images ( **F, G** ) shows multiple nodules ( *arrows* ) without any significant FDG uptake are seen in bilateral lungs, maximum nodule size of 0.6 × 0.4 cm in the right lung. These are stable on latter PET-CT ( **F** , **G** ).

In contrast, the spectrum of pulmonary metastasis on HRCT ranges over varying sizes, from discrete nodules to lymphangitic carcinomatosis. Following are the various patterns seen on radio-iodine scan correlated with other imaging findings:

1. Diffuse pattern of radioiodine uptake and its correlation with HRCT:
a. A 19-year-old young lady with pT4aN1bM1 differentiated thyroid carcinoma (DTC) with pulmonary metastases showed multiple tiny nodules in bilateral lungs with the maximum nodule size of 1.1 cm in the right lung upper lobe ( Fig. 2 ). The histopathology report (HPR) was indicative of classical papillary thyroid carcinoma (PTC) with extrathyroidal extension (ETE), perinodal invasion (PNI), and lymphovascular emboli (LVE). Her diagnostic ^131^ I scan showed unifocal neck uptake, diffuse tracer uptake in both the lungs, and a focal tracer uptake in the right lung upper zone corresponding to the nodule. Her stimulated serum Tg value at the time of studies was less than 300 ng/mL
b. A 38-year-old male patient presented with pT2N1bM1 classical papillary thyroid carcinoma (PTC) showed a few tiny nodules in the lungs on HRCT (maximum size of 2 mm, which were reported negative initially). His diagnostic ^131^ I scan showed diffuse iodine uptake in both the lungs ( Fig. 3 ); the initial stimulated serum thyroglobulin of 24.07 ng/mL at baseline and 0.03 ng/mL at follow-up scan. He had undergone three doses of radioiodine, with a cumulative dose of 602 mCi with persistently low serum Tg of 0.01 ng/mL during last follow-up.
c. A 9-year-old boy presented with neck swelling for 1 year. The CECT had demonstrated nodular 3 × 2.8 cm lesion in the bulky right thyroid lobe and bilateral cervical lymphadenopathy. The FNAC was suggestive of Bethesda category V PTC and metastatic PTC in the right neck node. He underwent total thyroidectomy with bilateral central compartment clearance and bilateral modified radical nodal dissection (II-V). His HPR was suggestive of classical PTC with multiple foci in the left lobe; extrathyroidal extension (ETE) and lymphovascular invasion (LVI) was present and there was involvement of bilateral lymph nodes with extranodal extension along with a deposit along the sympathetic chain. The diagnostic ^131^ I scan showed multiple foci of increased tracer uptake in the neck and diffuse uptake in bilateral lungs. SPECT-CT showed diffuse tracer uptake in the neck with focal increased uptake in a 4 mm nodule in the left lung lower lobe ( Fig. 4 ). His stimulated Tg was 141 ng/mL at the time of the scan. He was treated with 150 mCi RAI. The post-therapy scan showed multi-focal neck uptake with diffuse tracer uptake in both lungs.
2. Extent and Patterns of Focal Radioiodine Uptake and its correlation with HRCT
a. A 55-year-old male patient with widely invasive follicular carcinoma thyroid (FCT) presented with lung metastases. His initial diagnostic ^131^ I scan showed multi-focal tracer uptake in bilateral lungs ( Fig. 5 ). He had received with radioiodine multiple times with a cumulative dose of 782 mCi. The scan showed resolution of most of the lung lesions with faint tracer uptake seen in the lower lobe of left zones and a 0.8 cm nodule in the right lung on HRCT.
b. A 65-year-old female patient presenting with cough for 3 months, with FDG-PET/CT (pre-operative PET study and surgery done in a different institute) had shown bilateral hypermetabolic multiple lung metastasis, right supraclavicular lymph nodes, kidney lesion, metastatic marrow deposit in left iliac bone with multinodular goiter and serum thyroglobulin of 11,756 ng/mL. She underwent total thyroidectomy, right modified radical neck dissection, bilateral level VI and VII lymph node dissection in 2019. The histopathology was suggestive of follicular carcinoma of thyroid (FCT) involving the left thyroid lobe and metastatic carcinoma with follicular pattern, consistent with thyroid primary. The diagnostic ^131^ I scan showed increased uptake in bilateral lungs and left iliac region ( Fig. 6 ). She was treated with high-dose radioiodine in two sittings (cumulative dose of 397 mCi). Subsequently, she developed left cerebellar lesion after 8 months of therapy, underwent excision, and EBRT to the lesion until the last follow-up.
3. Negative Radioiodine scan in presence of Metastases in HRCT
a. A 39-year-old male patient presented with neck swelling and cervical lymphadenopathy for 1 year. He was diagnosed as pT3N1bMx papillary thyroid carcinoma (PTC) with high-risk factors, post total thyroidectomy, and bilateral neck nodal dissection. His diagnostic ^131^ I scan was suggestive of focal uptake in the neck with stimulated serum Tg of more than 300 ng/mL. He received 200 mCi of radioactive iodine therapy (RAI), followed by 173 mCi 7 months later for persistent neck uptake and elevated serum Tg levels. The follow-up ^131^ I scans were negative, with multiple bilateral lung nodules on HRCT ( Fig. 7 ). He was then diagnosed to have TENIS. FDG-PET/CT showed metabolically active scattered nodules in bilateral lungs, size up to 1.3 × 1.1 cm cervical and thoracic lymphadenopathy. He was later on considered for tyrosine kinase inhibitor (sorafenib) with suppressive thyroxine treatment. His follow-up FDG PET-CT 5 months later showed partial metabolic response.
b. A 49-year-old diabetic male patient with multifocal differentiated papillary thyroid carcinoma pT3N1BMx showed increased ^131^ I uptake in remnant thyroid, neck nodes, soft tissue near left shoulder, and faint uptake in bilateral lung nodules initially on diagnostic ^131^ I imaging ( Fig. 8 ). He underwent RAI therapy, cumulative of 647 mCi therapy in three sittings. His follow-up ^131^ I scan was only suggestive of faint focal uptake in the left shoulder with no uptake elsewhere with stimulated Sr. Tg greater than 300 ng/mL. FDG PET CT was suggestive of multiple hypermetabolic lesions in the left upper arm, tracheo-esophageal groove lesion, neck nodes, and multiple lung nodules, maximum size of 1 × 1 cm. He was then started on sorafenib but the patient defaulted for follow-up after a few months. His PET-CT 2 years later was suggestive of disease progression ( Fig. 8 ) and he was counseled to start on sorafenib again.
c. A 11-year-old girl presented with anterior neck swelling for 2 to 3 years. She underwent total thyroidectomy with bilateral cervical nodal dissection from levels II to V with central compartment clearance. The histopathology report was suggestive of papillary thyroid carcinoma with extrathyroidal extension, metastatic lymph nodes, and lymphovascular emboli. She was treated with 43 mCi radioiodine in a different center. Her post-therapy scan showed only intense uptake in neck ( Fig. 9A ). The follow-up diagnostic scan showed complete ablation of neck lesions with no ^131^ I avid lesion elsewhere but with persistently high serum Tg level of 26.24 ng/mL. Her FDG PET-CT with HRCT showed multiple tiny nodules without any significant uptake in bilateral lungs ( Fig. 9A–C ). The lesions were stable for 1 year ( Fig. 9D–G ).

### Treatment Response Assessment

#### Complete Response of Pulmonary Metastasis

**Fig. 10:**
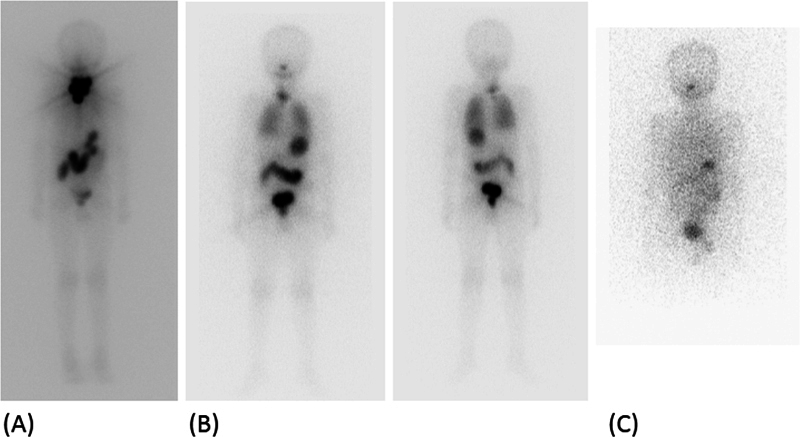
The first post-therapy image ( **A** ) showing intense uptake in the neck with low-grade/minimal diffuse tracer uptake in bilateral lungs. The follow-up scan ( **B** ) at 6 months following first therapy shows reduction in the intensity of the neck and presence of diffuse uptake in bilateral lung lesions. The last image ( **C** ) showed complete resolution of all the radiodine avid disease with normal HRCT.

**Fig. 11:**
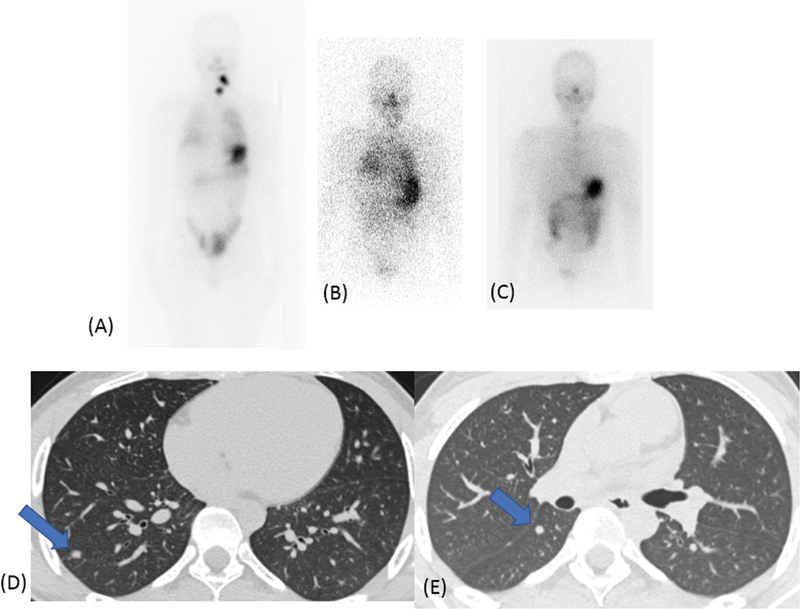
Initial ^131^ I scan ( **A** ) showing multifocal uptake in neck with diffuse increased uptake in bilateral lung parenchyma. His follow up scan after 2 RAI therapies (cumulative dose of 286 mCi) showed resolution of all ^131^ I avid disease ( **C** ). Suppressed serum Thyroglobulin (Tg) 1 year after complete response was 5.13 ng/ml. Therefore, HRCT ( **D, E** ) was advised and it showed sub-cm lung nodules ( *blue arrows* ).

a. A 10-year-old boy presented with metastatic papillary thyroid carcinoma to cervical nodes and lungs. His ^131^ I imaging showed cervical lesions and diffuse uptake in bilateral lungs ( Fig. 10 ) with stimulated serum Tg of 133 ng/mL. He was treated with multiple fractions of high-dose radioiodine (cumulative dose of 437 mCi). His follow-up ^131^ I imaging showed complete response with stimulated Tg of 0.3 ng/mL at last follow-up. HRCT at last follow-up was normal.
b. A 13-year-old boy presented with anterior neck swelling of 1 month duration and diagnosed with metastatic papillary thyroid carcinoma to cervical nodes. He underwent total thyroidectomy with bilateral central compartment clearance and bilateral modified neck dissection. The diagnostic ^131^ I scan showed multi-focal uptake in the neck and diffuse uptake in bilateral lungs ( Fig. 11 ) with stimulated Tg of 253 ng/mL. He was treated with 286 mCi ^131^ I in two doses 6 months apart. The stimulated Tg reduced to 32.2 ng/mL post-second therapy. The follow-up diagnostic ^131^ I imaging showed complete resolution of lung uptake 2 years later with stimulated Tg of 5.13 ng/mL. However, his HRCT at this point showed few sub-centimeter nodules in bilateral lungs, more on right side for which he was continued on thyroxine suppression.

#### Partial Response

**Fig. 12:**
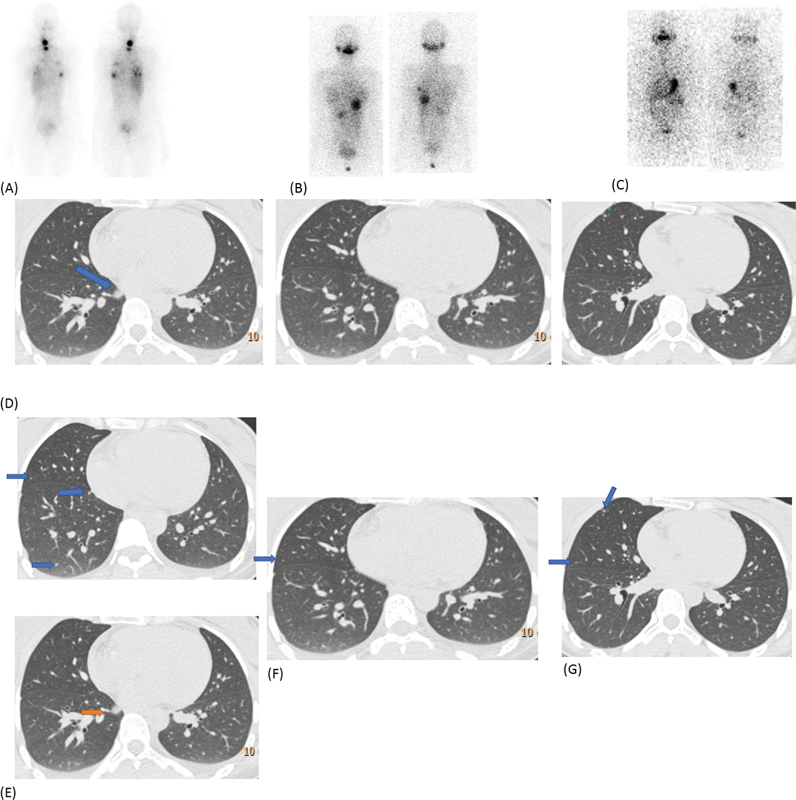
( **A** ) First post-therapy scan shows increased tracer uptake in the neck and focal pattern of tràcer uptake in bilateral lungs with sub-cm nodules, largest 0.8 cm in the right lung lower lobe ( *blue arrow* ). ( **B** ) After second radioiodine therapy, there is a significant resolution of most of the lesions ( *middle* ) and the follow up ^131^ I scan ( **C** ) showed near-complete response on both ^131^ I with the HRCT images illustrated. ( **D** ) Tiny nodules ( *blue arrows* ) seen at initial work up. ( **E** ) Largest nodule ∼ 0.8 cm in right lung lower lobe ( *orange arrow* ). ( **F** ) Resolution of the larger lesion but persistent tiny nodules ( *blue arrow* ) after first RAI therapy. ( **G** ) A few very tiny nodules ( *blue arrows* ) after second therapy as radioiodine scan shows complete response.

**Fig. 13:**
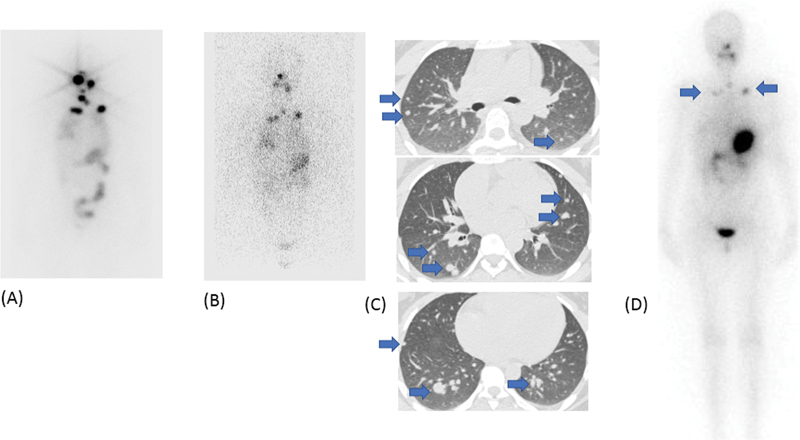
( **A** ) Initial diagnostic ^131^ I scan shows increased tracer uptake in the neck and focal tracer uptake in the lungs alongside diffuse pattern in lower zones. Follow-up scan after two doses of radioiodine therapy ( **B** ) shows persistent lung lesions and ablation of neck disease. HRCT images ( **C** ) showed multiple varying nodules in lungs, largest 1.4 cm in the right lung lower lobe. ( **D** ) Last post-therapy scan (after cumulative dose of 922 mCi) showed persistent radioiodine avid lung lesions in bilateral upper zones ( *arrows* ).

a. A 24-year-old lady presented with neck swelling of 4 years' duration and diagnosed with papillary thyroid carcinoma. She underwent total thyroidectomy with bilateral central compartment clearance and right-sided selective nodal dissection. Histopathology was suggestive of papillary thyroid carcinoma with cervical nodal metastases (pT3N1b), with perinodal extension. The ^131^ I diagnostic scan showed multi-focal neck uptake and in bilateral lung regions, predominantly in bilateral lower lobes ( Fig. 12 ) while her stimulated thyroglobulin (Tg) was 36.36 ng/mL. Her baseline HRCT showed a few sub-centimetre sized nodules in lower lobes of both lungs, largest 0.8 cm nodule in the right lung lower lobe. She received 378 mCi radio-iodine therapy in two divided doses. The follow-up ^131^ I scan 2.5 years later was normal and tiny nodule in the right lung middle lobe with stimulated Tg of 1.21 ng/mL. Other nodules had resolved on latest HRCT ( Fig. 12A–G ). She is currently under observation and doing well.
b. A 18-year-old young lady underwent total thyroidectomy with bilateral central compartment clearance, bilateral selective neck dissection for papillary thyroid carcinoma with regional nodal metastases. The diagnostic ^131^ I scan showed multi-focal neck and bilateral pulmonary foci of uptake ( Fig. 13 ). Her stimulated Tg at the time of first diagnostic scan was more than 300 ng/mL. She was treated with cumulative 922 mCi radioiodine in divided doses. Her HRCT showed multiple nodules, largest 1.4 cm in the right lung lower lobe. She is presently on suppressive treatment with unstimulated Tg of 6.3 ng/mL.

#### Progressive, Refractory Disease

**Fig. 14:**
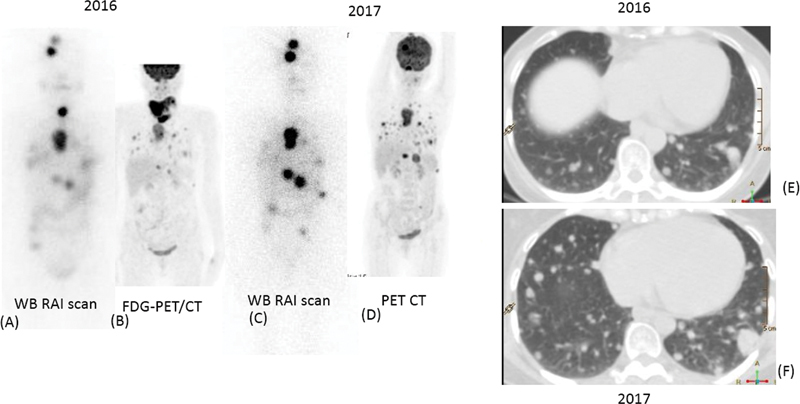
Initial ^131^ I scan ( **A** ) shows multiple skeletal, brain, adrenal, and lung lesions (focal pattern). After two doses of radio-iodine therapy, post-therapy scan shows resolution of only neck disease with new lesions on HRCT images ( **E, F** ) showing increase in the size of lung lesions.

**Fig. 15:**
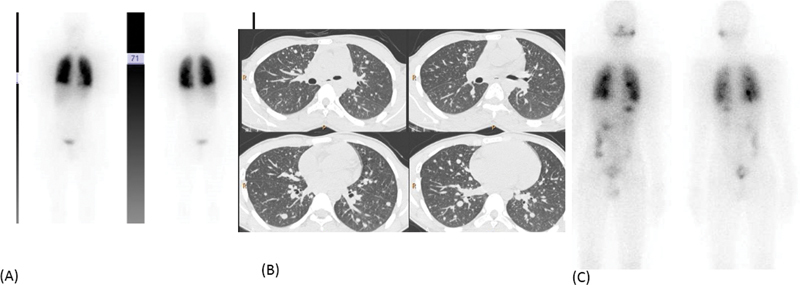
Diffuse pattern of lung metastasis is seen on initial ^131^ I images ( **A** ) with reticulo-nodular lesions on HRCT (maximum size of 1.1 cm) ( **B** , *left panel* ). After three doses of radio-iodine, there is persistent diffuse pattern of tracer uptake in the lungs ( **C** ), with marginal increase in size of few of the lung nodules ( **B** , *right panel* ).

**Fig. 16:**
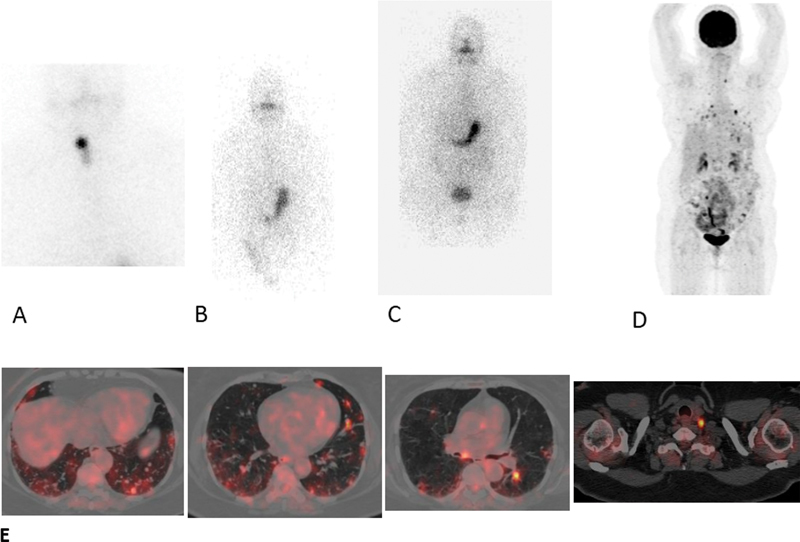
( **A** ) Initial diagnostic scan showing uptake only in the neck. ( **B** ) Follow-up radioiodine scan with no abnormal uptake. ( **C** ) Repeat radio-iodine scan with no ^131^ I-avid lesion. ( **D** ) FDG-PET/CT showing multiple metabolically active bilateral lung nodules and cervical lymph nodes. ( **E** ) Fused transaxial FDG-PET/CT images showing multiple bilateral lung nodules of maximum size of 0.6 cm with SUVmax. of 5.75; multiple hypermetabolic mediastinal and cervical nodes with SUVmax of 12.2.

a. A 55-year-old lady presented with metastatic thyroid carcinoma involving the lungs, bones, and adrenal gland at the time of presentation. She underwent total thyroidectomy and lymph node dissection. The histopathology was suggestive of poorly differentiated thyroid carcinoma, with ETE. Her initial HRCT chest showed scattered varying sized lung nodules, maximum up to 1.8 cm, which increased to 2.9 cm 1 year later. She underwent palliative EBRT to calvarial metastasis 1 month later followed by RAI 3 months later; palliative EBRT to sternal mass. She was started on sorafenib and treated with second high-dose RAI after 6 months. There was partial response on FDG PET CT after third high dose of RAI. However, she presented with disease progression 1 year later on PET-CT with new brain metastases ( Fig. 14 ). She was given brain RT and then she was referred for supportive care locally.
b. A 15-year-old boy with pT2N1bM1, stage II papillary thyroid carcinoma, had multiple lung nodules at the time of initial diagnosis. His stimulated serum Tg was 264 ng/mL in the beginning and increased to over 300 ng/mL throughout his follow-up. His baseline HRCT chest showed multiple scattered reticulo-nodular lesions, maximum size of 1.1 cm. He received a cumulative dose of 892 mCi RAI over 7 years in multiple divided doses with persistent disease ( Fig. 15 ).
c. A 64-year-old female patient was incidentally found to have thyroid nodules while being investigated for bronchiectasis. The CT scan was suggestive of infective lung changes. She was diagnosed with PTC on FNAC and underwent total thyroidectomy with lymph node dissection. Her HPR was suggestive of tall cell variant of PTC (pT3N1b). Her initial diagnostic radio-iodine scan showed focal tracer uptake in the neck with stimulated serum Tg of 140.13 ng/mL. She was treated with 150 mCi RAI. Her follow-up radio-iodine scan showed no abnormal uptake but with stimulated Tg value of 192.36 ng/mL. She was put on levothyroxine suppressive treatment for 2 years during, which time her TSH-suppressed Tg value was 1 ng/mL and she was asymptomatic during this period. Subsequently, she presented with cough and raised stimulated serum Tg value of 1,763 ng/mL, for which she underwent FDG-PET/CT. The PET-CT showed hypermetabolic bilateral lung nodules of maximum size 0.6 cm and SUVmax of 5.75; multiple hypermetabolic mediastinal and cervical nodes was noted with SUVmax of 12.2 ( Fig. 16 ). She was considered for tyrosine kinase inhibitors at this time.

## Discussion

Thus, in this essay, we have attempted to illustrate the varied clinical and imaging presentation of lung metastases from thyroid cancer. While diagnostic ^131^ I imaging is an efficient imaging modality in early and accurate detection of lung metastases, 8 especially on post-therapy scans, 9 there are certain scenarios where additional imaging are required for diagnosis, response, and disease assessment and prognostication.

Radioiodine non-avid metastasis are not rare as initially thought. Therefore, volumetric HRCT can have an incremental benefit in initial work-up of patients with high suspicion of distant metastases, 10 especially in the setting of raised serum thyroglobulin.

### Diagnosis

False positive uptake on whole body radioiodine scan can be observed and have been attributed to either physiologic (retention cysts, breast, and thymus); contamination on body surfaces; other pathologies (head, neck and chest infection or inflammation; other benign or malignant lesions such as lung, breast cancer); iatrogenic such as the suture granulomas or metallic implants. 11 As these various false positives are noted on radio-iodine scans and hence, concluding on planar imaging only should be meticulous. 9 11 12 TENIS and high-risk cases are frequently assessed with additional imaging such as CT or FDG-PET/CT for lesion detection and further characterization. 1 FDG-PET/CT has high accuracy of over 80% in detecting metastasis in TENIS cases along with high specificity, positive and negative predictive values. 13 14 15 Post-therapy SPECT-CT has been reported to add valuable information in TENIS patients demonstrating non-radioiodine avid anatomic lesions. 16

### Response Assessment

Iodine avid lung metastasis show complete response in 20 to 24% and partial response in approximately 50 to 60% of the patients. 17 18 However, radioiodine refractory (RAIR) and radioiodine negative cases will need additional imaging for deciding the management. Pulmonary metastasis from thyroid cancer largely have variable growth rate based up the tumor biology. Tumor volume doubling time (TvDT) is a parameter proposed by Sabra et al on the lines of thyroglobulin doubling time proposed by Miyauchi. 19 TvDT is a measure of growth rate. TvDT is calculated by taking two most prominent lesions on CT and measuring AP as well as transverse diameter. Those with TvDT of less than 1 year have poor prognosis while those with TvDT of 2 to 4 years are good candidates for multi-kinase inhibitors. 19 TvDT is a useful biomarker for risk stratification and prognosis. Rapidly progressive iodine refractory thyroid carcinoma was treated with multi kinase inhibitors showed improved disease-specific survival when TvDT increased. 20

### Prognostic Predictors in Pulmonary Metastasis

Categorization of lung metastases based on ^131^ I imaging is directly related to treatment efficacy while on morphological imaging is an overall prognostic marker. Pulmonary nodules of 1 cm or greater have less probability of complete remission. 21 It is also known that older age at diagnosis (≥ 40–55 years), radioactive iodine (RAI) non-avidity, radio-iodine refractoriness, pre-operative or late diagnosis of metastasis and macro-nodular metastasis (especially those with ≥ 1 cm) were predictive of decreased progression-free survival and disease-specific survival. 18 22 Therefore, such cases require stringent monitoring protocols especially in high-risk factor group. 23

FDG PET-CT parameters such as SUVmax, TLG, and MTV are associated with prognostic parameters such as disease progression and survival. 24 They additionally serve as objective tools of assessment while monitoring the lesions. 25 In the setting of elevated Tg but negative radioiodine scan; FDG PET-CT can give additional information on tumor burden. It was found that those who achieved good reduction in Tg levels on suppressive hormonal therapy and with negative FDG PET-CT had good outcome, showing high negative predictive value of FDG PET-CT. 26

## Conclusion

In summary, pulmonary metastases in thyroid carcinoma demonstrates varying imaging characteristics and disease biology that could be appropriately deciphered with a multi-modality patient-specific diagnostic approach and in certain situations would need multi-disciplinary management. Awareness about the atypical presentations helps in early identification as well as effective management. Larger prospective trials are required to address the unmet questions in long-term management of pulmonary metastases. We have to mention here that while HRCT of the lung as an added tool provides detailed visualization of the lung parenchyma, the routine adoption of SPECT-CT in patients with pulmonary metastases (in diagnostic or post-treatment settings) could provide equivalent or even incremental information from further management viewpoint. Further prospective comparative studies in this domain would be worthwhile for definitive conclusion.
